# Biofilm Formation in Methicillin-Resistant *Staphylococcus aureus* Isolated in Cystic Fibrosis Patients Is Strain-Dependent and Differentially Influenced by Antibiotics

**DOI:** 10.3389/fmicb.2021.750489

**Published:** 2021-10-15

**Authors:** Agathe Boudet, Pauline Sorlin, Cassandra Pouget, Raphaël Chiron, Jean-Philippe Lavigne, Catherine Dunyach-Remy, Hélène Marchandin

**Affiliations:** ^1^VBIC, INSERM U1047, Université de Montpellier, Service de Microbiologie et Hygiène Hospitalière, CHU Nîmes, Nîmes, France; ^2^HydroSciences Montpellier, Université de Montpellier, CNRS, IRD, Département de Microbiologie, CHU de Nîmes, Montpellier, France; ^3^VBIC, INSERM U1047, Université de Montpellier, Nîmes, France; ^4^HydroSciences Montpellier, Université de Montpellier, CNRS, IRD, Centre de Ressources et de Compétences de la Mucoviscidose, CHU de Montpellier, Montpellier, France

**Keywords:** cystic fibrosis, methicillin-resistance *Staphylococcus aureus* (MRSA), biofilm, antibiofilm activity, ceftaroline, ceftobiprole, linezolid

## Abstract

Cystic fibrosis (CF) is a genetic disease with lung abnormalities making patients particularly predisposed to pulmonary infections. *Staphylococcus aureus* is the most frequently identified pathogen, and multidrug-resistant strains (MRSA, methicillin-resistant *S. aureus*) have been associated with more severe lung dysfunction leading to eradication recommendations. Diverse bacterial traits and adaptive skills, including biofilm formation, may, however, make antimicrobial therapy challenging. In this context, we compared the ability of a collection of genotyped MRSA isolates from CF patients to form biofilm with and without antibiotics (ceftaroline, ceftobiprole, linezolid, trimethoprim, and rifampicin). Our study used standardized approaches not previously applied to CF MRSA, the BioFilm Ring test® (BRT®), the Antibiofilmogram®, and the BioFlux™ 200 system which were adapted for use with the artificial sputum medium (ASM) mimicking conditions more relevant to the CF lung. We included 63 strains of 10 multilocus sequence types (STs) isolated from 35 CF patients, 16 of whom had chronic colonization. The BRT® showed that 27% of the strains isolated in 37% of the patients were strong biofilm producers. The Antibiofilmogram® performed on these strains showed that broad-spectrum cephalosporins had the lowest minimum biofilm inhibitory concentrations (bMIC) on a majority of strains. A focus on four chronically colonized patients with inclusion of successively isolated strains showed that ceftaroline, ceftobiprole, and/or linezolid bMICs may remain below the resistance thresholds over time. Studying the dynamics of biofilm formation by strains isolated 3years apart in one of these patients using BioFlux™ 200 showed that inhibition of biofilm formation was observed for up to 36h of exposure to bMIC and ceftaroline and ceftobiprole had a significantly greater effect than linezolid. This study has brought new insights into the behavior of CF MRSA which has been little studied for its ability to form biofilm. Biofilm formation is a common characteristic of prevalent MRSA clones in CF. Early biofilm formation was strain-dependent, even within a sample, and not only observed during chronic colonization. Ceftaroline and ceftobiprole showed a remarkable activity with a long-lasting inhibitory effect on biofilm formation and a conserved activity on certain strains adapted to the CF lung environment after years of colonization.

## Introduction

Cystic fibrosis (CF) is a recessive genetic disease linked to a mutation in the cystic fibrosis transmembrane conductance regulator (*cftr*) gene encoding for an ion channel whose dysfunction causes thickening of the respiratory mucus in CF patients (Rowe et al., 2005). The resulting dehydrated, sticky mucus provides an ideal environment for bacterial colonization, and CF patients are therefore particularly exposed to pulmonary infections from an early age. Among the prevalent opportunistic pathogens in CF worldwide, *Staphylococcus aureus* is generally the most commonly identified microorganism (Registre français de la mucoviscidose - Bilan des données, 2019; The Cystic Fibrosis Foundation Patient Registry, 2019; Zolin et al., 2020). *S. aureus* is acquired early on in the life of CF patients, and from the age of 5years, *S. aureus* will colonize or infect approximately 70% of children. Infections may be persistent in certain patients, and in 2018 in Europe, the prevalence of chronic *S. aureus* in CF patients was 35.68% (34.34% in children; 37.12% in adults; Zolin et al., 2020). Multidrug-resistant *S. aureus* strains, known as methicillin-resistant *S. aureus* (MRSA), are of particular concern. A degree of variation in MRSA prevalence is observed between countries for the CF population with a lower prevalence observed in Europe (e.g., 5.9% in France in 2019; Registre français de la mucoviscidose - Bilan des données, 2019) compared with the United States where the widespread distribution of community-acquired MRSA clones like the USA300 clone (Glikman et al., 2008; Goss and Muhlebach, 2011) is associated with a 25% rate of CF patients with at least one MRSA isolate in 2019 (The Cystic Fibrosis Foundation Patient Registry, 2019). In Europe, the epidemiology of circulating MRSA clones in the CF population remains largely unexplored. MRSA eradication in CF patients is currently recommended as compared with methicillin-susceptible *S. aureus* (MSSA), MRSA persists longer in the airways of these patients, resulting in more serious pulmonary dysfunction and lung damage with a greater decline in mean forced expiratory volume in 1s (FEV1) and in greater use of antibiotics (Ren et al., 2007; Dasenbrook et al., 2008; Vanderhelst et al., 2012). However, MRSA eradication is challenging, not only due to the antimicrobial multidrug resistance patterns of the strains but also to the diverse bacterial adaptation pathways developed in the CF lung environment, including biofilm formation which represents an important barrier to eradication treatment and host defenses, thus contributing to bacterial persistence in the CF lung (Goerke and Wolz, 2010; Hirschhausen et al., 2013). Identifying antimicrobial treatments that may limit biofilm formation has become a matter of concern in the management of numerous infections, particularly those involving *S. aureus* which is a well-known pathogen causing diverse biofilm-associated infections other than those encountered in CF, such as medical device-associated and catheter-related infections, and bone infections (Miquel et al., 2016), and over the years, studies have aimed to evaluate the antibiofilm activity of various antimicrobial agents (antibiotics as well as antimicrobial peptides and natural substances). These studies underline the limitations of *in vitro* studies of bacterial biofilms, particularly the poor reproducibility of certain methods and the influence of cultivation conditions. Considering these limitations, a variety of protocols and methods for *in vitro* drug activity testing against staphylococcal biofilms has been successively introduced with the aim of standardizing protocols and improving inter-laboratory reproducibility, including microfluidics systems which more closely approximate natural biofilms (Benoit et al., 2010; Guzmán-Soto et al., 2021).

In this context, the main objective of our study was to evaluate the effects of antibiotics on the biofilm formation of MRSA isolates from CF patients. Our study focused on trimethoprim, rifampicin, and linezolid which are some of the drugs that may be used to deal with MRSA infections in CF patients (Fusco et al., 2015; Zobell et al., 2015; Akil and Muhlebach, 2018). Two broad-spectrum cephalosporins (more recently approved for the treatment of bacterial pneumonia due to MRSA), ceftaroline and ceftobiprole, were also evaluated. Our study was based on standardized approaches not previously applied to CF MRSA isolates, that is, the BioFilm Ring test® (Chavant et al., 2007; Ren et al., 2007) and the microfluidics system BioFlux™ 200 (Benoit et al., 2010) which were adapted for use with the artificial sputum medium (ASM), a mucin-containing synthetic growth medium (Sriramulu et al., 2005; Dinesh, 2010) to test MRSA biofilm formation, and antibiotic efficacy against biofilm formation in conditions more relevant to the CF lung. The BRT® is used to quickly evaluate bacterial adhesion and early biofilm formation. The BRT® has also been proposed as a tool for evaluating the capacity of antibiotics to inhibit biofilm formation through an approach called the Antibiofilmogram® (Tasse et al., 2016), also used in our study. Finally, we used the BioFlux™ 200 continuous flow system to study biofilm formation in dynamic conditions. Strains studied were clinical isolates genotyped by multi locus sequence typing (MLST) and, for selected strains, by whole-genome sequencing (WGS). Due to the highly complex patterns of bacterial colonization in CF patients’ airways, we also included strains that were co-isolated from within a sample or successively isolated from chronically colonized patients with the aim of bringing new insights into the behavior of strains adapted to the CF lung during persistent colonization.

To the best of our knowledge, studies like this have never been performed before and our study therefore provides original results on comparative biofilm formation with and without antibiotics for a collection of clinically and genetically documented MRSA from CF patients.

## Materials and Methods

### Patients, Strains, and Study Design

**Figure 1:**
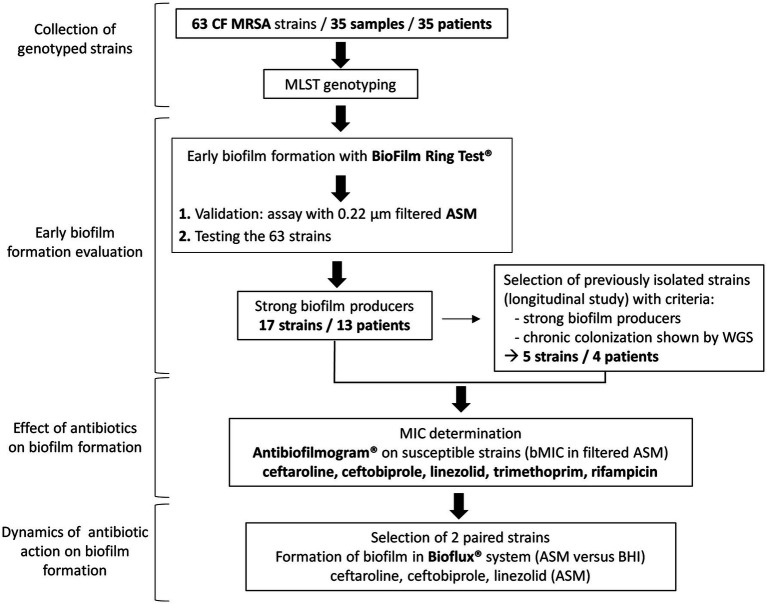
Flowchart showing the main steps of the study. CF, cystic fibrosis; MRSA, methicillin-resistant *Staphylococcus aureus*; MLST, multilocus sequence typing; ASM, artificial sputum medium; WGS, whole-genome sequencing; MIC, minimum inhibitory concentration; bMIC, biofilm MIC; and BHI, brain heart infusion medium.

Methicillin-resistant *S. aureus* strains included in this study were isolated from routinely sampled sputum specimens in patients attending the CF center at Montpellier University Hospital, France, and analyzed as part of their standard follow-up. Strains were recovered in 35 patients representing all patients with MRSA isolation in our center over a 9-year period. Strains had been stored at −80°C. These strains were studied for their ability to form biofilm in the presence or absence of antibiotics according to the steps presented in the flow diagram in Figure 1. Briefly, we first identified strains that were strong biofilm producers among a collection of clinically documented and genetically characterized CF MRSA using the BRT® and the ASM. Strong biofilm producers were then included in the evaluation of antibiotic efficacy against biofilm formation using the Antibiofilmogram® method. Selected isolates were then used to conduct: (i) a longitudinal analysis of the evolution of antibiotic efficacy on biofilm formation over time in chronically colonized patients (selection of clonal strains successively isolated in four patients) and (ii) a study of the dynamics of biofilm formation under flow in the absence and presence of antibiotics (selection of two paired strains successively isolated in a chronically colonized patient).

### Multilocus Sequence Typing

All strains were cultured on Trypticase Soy Agar with 5% Sheep Blood (bioMérieux, Marcy l’Étoile, France), and one colony was subcultured on the same agar medium for further analysis. DNA extraction was performed as previously described (Predari et al., 1991). The amplification of the seven housekeeping genes included in the MLST scheme for *S. aureus* was performed as previously described (Enright et al., 2000; Crisóstomo et al., 2001). Sequencing was done on a 3500xL Genetic Analyser (Thermo Fisher Scientific Waltham, Massachusetts, United States), and sequence analysis was performed using SeqScape Software v3.0™ from the same company. The sequence type (ST) was determined for each strain *via* the PubMLST database (Jolley et al., 2018)^1^ or after whole-genome sequencing (wgMLST).

### Whole-Genome Sequence Analysis

Whole-genome sequencing analysis was performed for the 11 CF MRSA isolates (five “early” isolated strains and six “late” isolated strains in four chronically colonized patients) selected for the longitudinal analysis of the evolution of antibiotic efficacy on biofilm formation over time to prove that the MRSA persistence in these patients was truly a chronic colonization by a clonal strain. For six of these strains, WGSs were available from previous work (Boudet et al., 2021); for the five remaining strains, WGSs were obtained by an Illumina MiSeq platform (Illumina Inc., San Diego, CA, United States) and analyzed as previously described (Boudet et al., 2021). Regarding analysis of single nucleotide polymorphisms (SNPs), SNPs were called using Snippy (Seemann, 2015) and SNPs numbers were interpreted according to the criteria of Ankrum and Hall (2017) which define strains with ≤71 SNPs as the “same” strains, strains with 72–123 SNPs as “very closely related” strains, strains with 124–156 SNPs as “closely related” strains, and strains with ≥157 SNPs as “distantly related” strains.

DDBJ/ENA/GenBank accession numbers for the 11 WGS are JAGPWS000000000, JAGPWY000000000 and JAGPWZ000000000 (strains 5.1, 5.2, and 5.3 in Patient 5); JAGPWI000000000, JAGPWQ000000000 and JAGPWR000000000 (strains 6.1, 6.3, and 6.4 in Patient 6); JAHXBM000000000, JAHXBL000000000, and JAHXBK000000000 (strains 17.2, 17.3, and 17.6 in Patient 17); JAHXBJ000000000 and JAHXBI000000000 (strains 18.1 and 18.2 in Patient 18).

### Culture Media and Growth Rates

After initial culture of frozen strains on Trypticase Soy Agar with 5% Sheep Blood (bioMérieux, Marcy l’Étoile, France), we used different media according to the test performed. Mueller-Hinton broth was used as the broth microdilution reference method to determine minimum inhibitory concentrations (MIC) of antibiotics. The ASM, a medium mimicking the sputum found in the respiratory tract of CF patients (Sriramulu et al., 2005; Dinesh, 2010), was used to evaluate biofilm formation and antibiotic efficacy against biofilm formation in conditions more relevant to the CF lung using the different approaches of this study and developed below: the Biofilm ring Test® (BRT®), the Antibiofilmogram®, and the continuous flow system BioFlux™ 200. However, the brain heart infusion (BHI) medium is recommended by the BRT® manufacturer and was therefore used with ASM for comparative purposes. As the opacity of the ASM did not provide a compatible image with the BRT® reading software, 0.22μm-filtered ASM was compared to BHI medium on selected isolates with the BRT®. BHI medium was also compared with ASM in the assays conducted on the BioFlux™ system. According to the medium used for the assay, strains were pre-cultured for 24h in the same medium, BHI broth, or ASM. Due to the use of these three culture conditions, growth properties of selected strains in BHI broth, ASM, and 0.22μm-filtered ASM were compared. Overnight cultures at 37°C were inoculated into the same fresh medium [identical dilution factor for all three conditions to obtain a suspension of optical density (OD) at 600nm (OD_600_) of 0.1], and incubation was carried out at 37°C with agitation for 24h. Cell growth was monitored over 24h through colony-forming unit (CFU) counts with eight data points: 0, 0.5, 1, 2, 3, 4, 6, and 24h. For CFU counts, cultures were diluted serially in the medium and plated on Trypticase Soy Agar with 5% Sheep Blood plates which were incubated for 24h at 37°C. All assays were performed in duplicate.

### Bacterial Adhesion and Biofilm Formation Assessment Using Biofilm Ring Test®

The BRT® based on the measurement of the mobility of superparamagnetic microbeads subjected to a magnetic field was used to study the early stages of biofilm formation according to the manufacturer’s recommendations (Chavant et al., 2007). Briefly, standardized bacterial cultures were incubated at 37°C in 96-well microtiter plates in the presence of magnetic beads. At set time-points, the plates were placed on a magnetic block and put in the reader. The images of each well before and after magnetic attraction were analyzed with the BioFilm Control software (BFC Elements® 3), which gives a biofilm formation index (BFI). The adhesion ability of each strain was expressed as this BFI that is inversely proportional to the attached cell number. A high BFI value indicates high bead mobility under magnetic action that corresponds to the absence of biofilm formation (a spot is visible due to bead accumulation above the mini-magnet), while lower values reflect partial to complete immobilization of beads due to the bacteria embedded in a biofilm (no visible spot for complete bead immobilization). Two incubation times were defined: 1.5 and 4h. Three replicate wells were performed per strain and per incubation time in three (BHI/filtered ASM comparison) or two independent experiments (tests in filtered ASM only). A negative control was systematically included in each experiment corresponding to the medium and beads without bacterial suspension. BFI values were calculated for each well, ranging from 0 (total bead immobilization, i.e., strongly adherent cells/strong biofilm formation) to 20 (no bead aggregation, i.e., non-adherent cells/no biofilm formation in the experiment conditions).

### Susceptibility Testing

Minimum inhibitory concentrations of ceftaroline, ceftobiprole, linezolid, rifampicin, and trimethoprim were determined for all CF MRSA strains using the microdilution method in Mueller-Hinton broth. According to the interpretative criteria of the European Committee on Antimicrobial Susceptibility Testing (EUCAST), strains were classified as susceptible (including “susceptible” and “susceptible, increased exposure” categories) or resistant (European Committee on Antimicrobial Susceptibility Testing, 2021). Serial 2-fold dilutions of antibiotics were as follows: 0.125–16mg/L for ceftaroline, 0.25–32 for ceftobiprole, 0.5–64mg/L for linezolid, 0.015–2mg/L for rifampicin, and 0.25–32 for trimethoprim. The reference strain *S. aureus* ATCC 29213 was used to monitor test performance.

The minimum biofilm inhibitory concentrations (bMIC) were determined using the Antibiofilmogram® test (BioFilm Control; Tasse et al., 2016). A schematic representation of the Antibiofilmogram® principle is available in Olivares et al. (2020). The test was performed using 0.22μm-filtered ASM for initial bacterial growth and suspension preparation. The microplates containing bacteria, magnetic beads, and antibiotics (20μl of antibiotic solutions) were incubated at 37°C for 4h before visual reading. The bMIC was determined for each antibiotic as the lowest concentration at which a spot corresponding to free beads attracted to the center of each well during magnetization, similar to negative control (0.22μm-filtered ASM and magnetic beads), was visible. A well without antibiotics filled with the bacterial suspension in 0.22μm-filtered ASM and magnetic beads was used as the positive control (absence of spot due to bead immobilization in biofilm). All assays were performed in duplicate.

### Biofilm Formation Assessment Using the BioFlux™ 200 System

The kinetics of biofilm formation and quantification in the absence and presence of antibiotics (ceftaroline, ceftobiprole, and linezolid) were assessed with the BioFlux™ 200 microfluid system as previously described (Naudin et al., 2019) with two different media, BHI medium and ASM. In this system, biofilm formation is studied under dynamic and controlled flow conditions and is followed by light microscopy in microfluidic wells (Benoit et al., 2010). The system consists of a 48-well plate with a microchannel connection between 24 pairs of two types of wells, an input well and an output well. Bacteria were grown overnight in each media. Bacterial suspensions with an OD_600_ of 0.1 +/− 0.05 after two 200th serial dilutions from the overnight culture were prepared in both media. To quantify biofilm formation, we prepared BioFlux system by adding 500μl of medium into the input well with a pressure of 1 dyne/cm^2^ for 10min. At the end of the 10min, the medium remaining in the input well was removed. Bacterial suspensions were then added in the input well for 36h of incubation at 37°C in BHI medium and ASM with a pressure of 0.2 dyne/cm^2^. The bacterial suspension was either added alone or in the antibiotic solution, i.e., last dilution performed in the antibiotic solution to give final concentrations of bacteria of 10^3^CFU/ml and antibiotic corresponding to the bMIC determined by the Antibiofilmogram® either in BHI broth or in filtered ASM. Biofilms were grown in monoculture, and the quantification of biofilm formation was done at 5, 12, 24, and 36h post-inoculation in the absence of antibiotics and at 5, 12, and 36h post-inoculation in the presence of antibiotics. Experiments were performed in duplicate. Biofilms were visualized with a Leica DM IRB inverted fluorescence microscope coupled with a CoolSNAP FX black and white camera (Roper Scientific, Trenton NJ, United States). ImageJ® software was utilized to calculate biofilm percentage. The 16-bit grayscale images were adjusted with the threshold function to fit the bacterial structure and were analyzed using the “Analyze Particles” function to calculate biofilm percentage in the microfluidic channel.

### Statistics

Results are presented as the mean±standard deviation. Statistical analyses were made using a t test in version 7 of GraphPad Prism (comparison of biofilm formed according to the condition, either medium or incubation time, in the BioFlux™ analyses) or using a Mann-Whitney U test in version 3.5.2 of R software (comparison of BFI values in BHI medium and filtered ASM). A value of *p*≤0.05 was considered to reflect significance.

## Results

### Patients and MRSA Collection Characteristics

**Figure 2:**
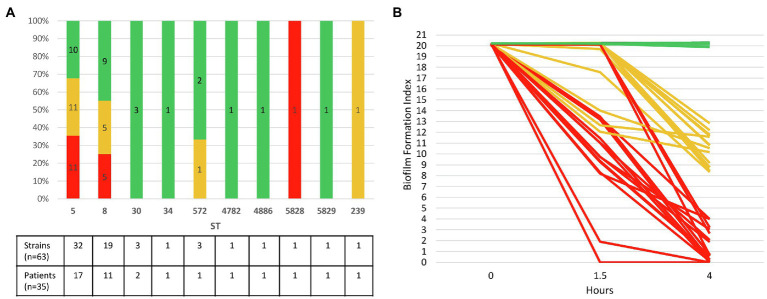
Ability of 63 CF MRSA strains to form early biofilm according to MLST genotype. **(A)** Relative distribution of strains according to their genotype MLST (ST, sequence type) and their ability to form early biofilm as shown in **(B)**. The number of strains and patients are indicated at the bottom of the figure for each ST. **(B)** Dynamics of early biofilm formation determined using BRT® and filtered ASM after 1.5 and 4h of incubation. Red, strains with low biofilm index (BFI) values (≤4) associated with full immobilization of the magnetic beads due to complete biofilm formation; green, non-adherent strains (BFI values: 19.9–20.3); orange, strains with intermediate BFI values (8.4–13.5).

The 35 CF patients (54.3% of female) had a mean age of 22.1years at inclusion in the study (range: 2–52years). Sixteen of them fulfilled the criteria for chronic colonization (>50% of respiratory samples collected during the last 12months positive for MRSA with at least four samples collected during that period; Zolin et al., 2020). The 63 strains had been isolated between 2007 and 2016 from 35 sputum samples in these patients (2–4 colonial morphotypes visually distinct observed during the routine bacteriological analysis of sputum samples recovered from 18 samples in 18 patients were included; Supplementary Table). MLST genotyping showed that the 63 strains belonged to 10 STs (Figure 2A). MRSA of ST5 (50.8% of strains and 48.6% of patients) and ST8 (30.2% of strains and 31.4% of patients) were the most frequent representing 81% of the strains (51 strains/63) and being identified in 80% of the patients (28 patients/35). Two patients had co-colonizations with strains from different STs within the same clonal complex (CC) identified in one sample [ST5 and ST4782 (CC5) in Patient 14, ST8 and ST5829 (CC8) in Patient 33]. In these cases, both genotypes identified differed by one (strains in Patient 14) or five mutations (strains in Patient 33) in one of the seven genes of the MLST scheme (*aroE* gene and *gmk* gene, respectively).

### Use of Artificial Sputum Medium to Test Bacterial Adhesion Using the Biofilm Ring Test®

Brain heart infusion medium is recommended by the BRT® manufacturer. However, the ASM containing mucin, amino acids, and free DNA was formulated to mimic the sputum of CF patients and its use is thought to be more representative of the lung conditions during CF. We therefore aimed to evaluate the use of ASM in the Biofilm Ring Test®, either to characterize the adhesion pattern and ability to form biofilm of clinical CF MRSA (BFI determination) or to perform an Antibiofilmogram® (bMIC determination). ASM is a turbid medium which yields invalid results in the BRT® but using ASM filtered through a 0.22μM filter system made it possible for the system to calculate the BFI. To go further with the use of filtered ASM, we compared the growth ability in BHI, ASM, and filtered ASM of three selected strains that were further categorized as strong biofilm producers: strains 5.3 and 6.4 that were also used for the comparison of the BRT® use in BHI medium and ASM, and strain 17.6 that was later studied using the BioFlux™ 200 system. Similar growth rates were observed in the three media for these three strains (Supplementary Figure 1). We then compared both media for BRT® use by comparing BFI values measured after 4h of incubation in BHI medium and filtered ASM for strains 5.3 and 6.4 (Supplementary Figure 2). Differences in adhesion patterns were observed depending on the medium used, with lower BFI values observed in filtered ASM compared with BHI medium and a significantly more pronounced adherence behavior in ASM compared with BHI medium for strain 5.3 (value of *p*<0.001 by Mann-Whitney U test). On the whole, these observations led us to consider the use of filtered ASM for further experiments using the BRT®.

### Evaluation of Early Biofilm Formation Using Biofilm Ring Test® in Filtered ASM

Biofilm formation index values evaluating the CF MRSA adhesion ability were distributed in three clearly separated groups after an incubation time of 4h: BFI values≤4, BFI values comprised between 8.37 and 13.52, and BFI values ranging from 19.87 to 20.31 (Supplementary Table; Supplementary Figure 3). We found that 27% of strains (17/63) isolated in samples from 37.1% of the patients (13/35) formed a strong biofilm after 4h of incubation in filtered ASM (BFI<4), including two strains of ST5 (strains 17.6 and 30.1) which were strongly adherent after 1.5h of incubation (Figure 2B). Nearly all the strains (94.1%, 16/17) that were early biofilm producers belonged to ST5 and ST8. It is worth noting that intra-sample heterogeneity in the ability to form biofilm was observed, strong biofilm-producing strains being co-isolated with strains displaying distinct ability to form biofilm in samples from five patients (38.5%, 5/13). A striking example was observed for Patient 25 for whom strong biofilm producers and non-adherent strains coexisted in a sample (Supplementary Table). Half of chronically colonized patients (8/16) were colonized by such strains with a strong capacity for biofilm production (Supplementary Table). Non-adherent strains represented 44.4% of the strains and were isolated in 18 patients (BFI: 19.87–20.31, as observed for the negative control that contained filtered ASM and beads only). Finally, 28.6% of the strains, isolated from 13 patients, with intermediate BFI values (8.37–13.52), were likely slower biofilm formers, and characterization of their adhesion kinetics would have required a longer incubation time (Supplementary Table; Supplementary Figure 3).

No obvious relation was observed between the ability to form early biofilm of the MRSA strains and the co-colonizers, particularly *Pseudomonas aeruginosa*. Out of the 63 strains/35 patients of the study, 40 MRSA strains were isolated in 21 patients with chronic colonization by *P. aeruginosa*. These strains were distributed in the three groups of strains defined according to their ability to form early biofilm (13 strains showed BFI≤4, 17 strains were non-adherent and 10 had intermediate BFI values).

### Antimicrobial Susceptibility of the 17 Strongly Adherent Strains

Determination of the MICs of the five antibiotics under consideration in this study showed that 11 strains (64.7%) were resistant to rifampicin, and three strains (17.6%) were resistant to linezolid, while the 17 strains were susceptible to trimethoprim, ceftobiprole, and ceftaroline (Table 1). bMICs were then determined using the Antibiofilmogram® approach in filtered ASM. Highly distinct patterns of bMICs were observed according to the antimicrobial agent (Table 1). No strains displayed bMIC for trimethoprim below the EUCAST threshold for resistance at 4mg/L (bMIC range: 8–>32mg/L) showing that this antibiotic was totally inefficient on biofilm formation initiation. The lowest bMICs were observed for ceftaroline and ceftobiprole with efficacy in preventing biofilm formation observed for 76.5 and 70.6% of strains, respectively (bMICs below the respective resistance breakpoint of 1 and 2mg/L) compared with linezolid (64.7% of strains with bMICs≤4mg/L) and rifampicin (17.6% of strains with bMICs≤0.5mg/L). Individual results for each of the 17 strains under evaluation are presented in Table 2. The capacity of rifampicin and linezolid to prevent biofilm installation was also evaluated for the sole strains that were susceptible in planktonic cultures; linezolid was able to inhibit biofilm formation of 78.6% of the susceptible strains (three out of 14) and rifampicin prevented biofilm installation of 50% of the susceptible strains (three out of six; Table 2).

**Table 1:** Overall susceptibility results for the 17 CF MRSA strains that were classified as strongly adherent/strong biofilm producers according to the Biofilm Ring Test® assay.

Antimicrobial agent	Minimal inhibitory concentration (MIC in mg/L)				Minimal biofilm inhibitory concentration (bMIC in mg/L)			
	Range	MIC_50_	MIC_90_	Resistant (%, *n*)	Range	bMIC_50_	bMIC_90_	Above the threshold for resistance (%, *n*)
Ceftaroline	<0.125–0.25	0.125	0.25	0	<0.125–4	0.25	4	23.5 (*4*)
Ceftobiprole	<0.25	<0.25	<0.25	0	<0.25–16	2	8	29.4 (*5*)
Linezolid	<0.5–32	1	16	17.6 (*3*)	<0.5–>64	2	>64	35.3 (*6*)
Rifampicin	<0.015–2	2	2	64.7 (*11*)	<0.015–>2	>2	>2	82.4 (*14*)
Trimethoprim	<0.25–2	0.5	2	0	8–>32	>32	>32	100 (*17*)

European Committee on Antimicrobial Susceptibility Testing (EUCAST) breakpoints for resistance were as follows: ceftaroline, 1mg/L; ceftobiprole, 2mg/L; linezolid, 4mg/L; trimethoprim, 4mg/L, and rifampicin, 0.5mg/L.

**Table 2:** Detailed susceptibility results for the 17 CF MRSA strains that were classified as strongly adherent/strong biofilm producers according to the Biofilm Ring Test® assay.

Patient	Strain	ST	BFI	Ceftaroline		Ceftobiprole		Linezolid		Rifampicin		Trimethoprim	
				MIC	bMIC	MIC	bMIC	MIC	bMIC	MIC	bMIC	MIC	bMIC
1	1.1	8	4	<0.125	2	<0.25	16	<0.5	16	2	>2	1	>32
5	5.2	5	0.12	<0.125	<0.125	<0.25	<0.25	32	>64	2	>2	0.5	>32
	5.3	5	0.75	<0.125	4	<0.25	8	16	32	2	>2	1	>32
6	6.3	5	0.58	<0.125	0.25	<0.25	2	1	16	2	>2	1	>32
	6.4	5	0.25	0.25	0.25	<0.25	<0.25	16	>64	2	1	1	>32
7	7.2	5	0.71	0.25	0.25	<0.25	2	<0.5	2	0.5	>2	0.5	8
17	17.6	5	0	<0.125	4	<0.25	4	<0.5	2	2	>2	0.5	>32
18	18.2	8	3.03	<0.125	0.5	<0.25	2	1	1	2	>2	1	8
20	20.1	5828	0.66	<0.125	4	<0.25	4	1	64	2	>2	2	>32
25	25.5	5	4	0.25	0.25	<0.25	<0.25	2	2	<0.015	<0.015	2	32
	25.6	5	3.22	<0.125	0.25	<0.25	8	4	4	<0.015	>2	0.5	>32
26	26.1	8	2.08	<0.125	<0.125	<0.25	2	<0.5	2	<0.015	0.015	<0.25	>32
28	28.3	5	0	<0.125	1	<0.25	2	<0.5	<0.5	2	>2	<0.25	>32
	28.4	5	0.25	<0.125	<0.125	<0.25	<0.25	<0.5	1	<0.015	<0.015	<0.25	8
30	30.1	5	0	<0.125	<0.125	<0.25	<0.25	<0.5	1	<0.015	>2	1	32
31	31.2	8	2.68	<0.125	<0.125	<0.25	0.5	<0.5	1	2	>2	0.5	>32
32	32.1	8	1.89	<0.125	<0.125	<0.25	<0.25	1	1	2	>2	0.5	>32

Minimum inhibitory concentrations (MIC) and biofilm MIC (bMIC) in mg/L. MICs were determined according to the reference dilution method. bMICs were determined in filtered ASM. Gray cells indicate MIC and bMIC values above the EUCAST breakpoints for resistance (indicated in footnotes of Table 1). ST, sequence type. BFI, biofilm formation index.

For three strains (strains 1.1, 5.3, and 20.1), bMICs of all the five antibiotics were above the corresponding resistance breakpoint and these strains were excluded from further analysis conducted to compare the efficacy of the various antimicrobial agents on biofilm formation by CF MRSA. Analysis was, thus, restricted to susceptible strains (planktonic cultures) that displayed at least one bMIC below the threshold for resistance (*n*=14; Table 2). Under these conditions, bMICs for ceftaroline were below the resistance threshold for 13 strains, bMICs for ceftobiprole were below the resistance threshold for 12 strains, and bMICs for linezolid were below the resistance threshold for 11 strains. A limited effect was observed for rifampicin with three strains out of 14, isolated from three patients, showing low bMIC values<0.015mg/L. However for two patients, these strains were associated either with a resistant strain (Patient 28) or with a strain for which the formation of biofilm was not inhibited by rifampicin (Patient 25) thereby making the drug of low interest considering its global effect on biofilm formation inhibition. More generally, intra-sample heterogeneity with coexistence of adaptive variants displaying distinct bMICs was observed for three out of the four patients for whom the analysis of multiple strains from a sample was performed (Patients 5, 25, and 28; Table 2).

### Study of Clonally-Related, Longitudinally-Isolated Strains in Four Patients

**Figure 3:**
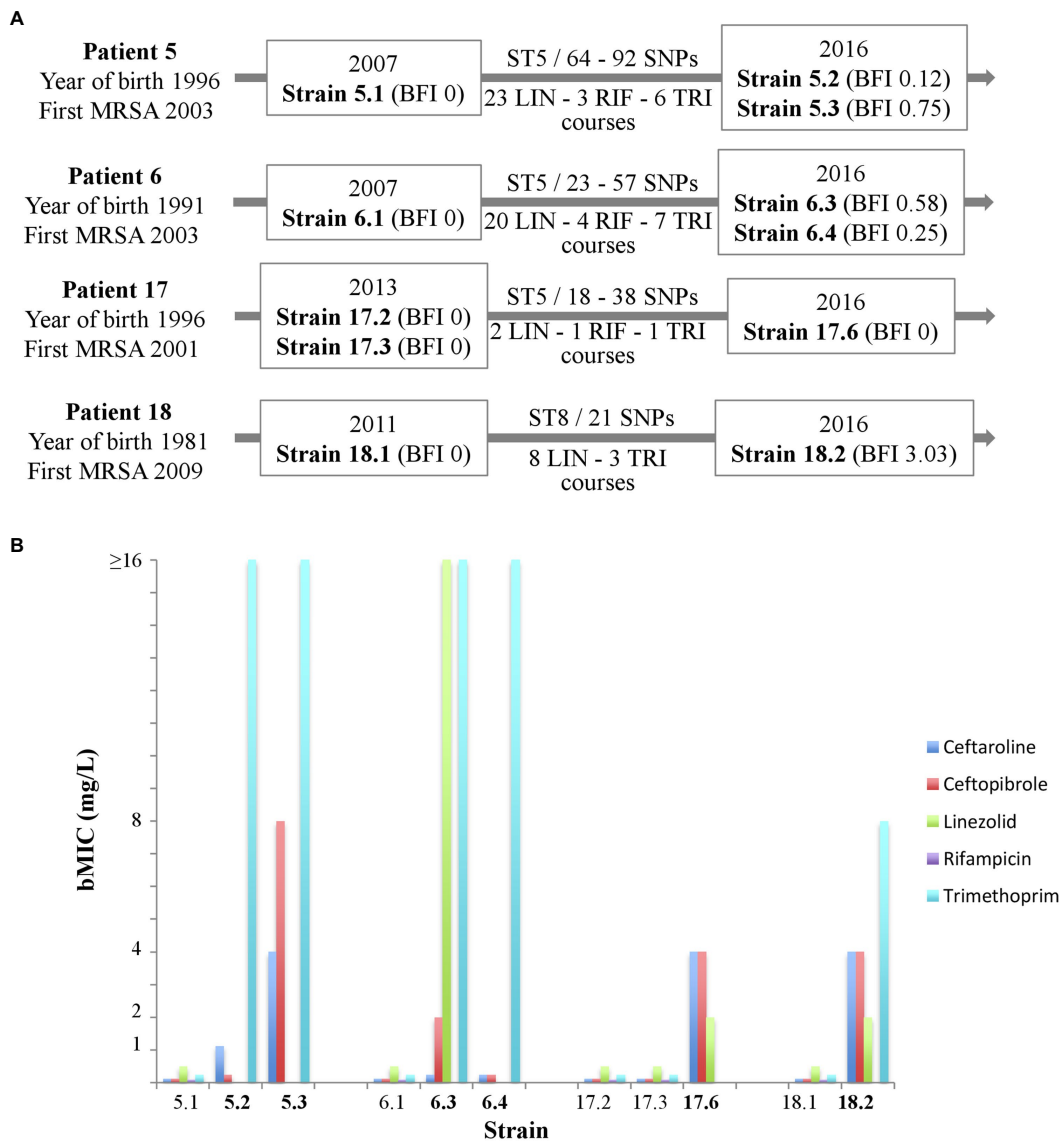
Antibiofilmogram® results for successively isolated MRSA strains in four cystic fibrosis patients (5 « early » strains; 6 « late » strains). **(A)** Schematic presentation of patients (designation, year of birth, and year of first colonization by MRSA), strains [year of isolation, strain designation, biofilm formation index (BFI) value, ST, single nucleotide polymorphism (SNP) number between successively isolated strains], and number of antimicrobial courses (LIN, linezolid; RIF, rifampicin; and TRI, trimethoprim). **(B)** Biofilm minimal inhibitory concentration (bMIC, mg/L) in 0.22μm-filtered artificial sputum medium of five antibiotics for the 11 strains included (data are presented for strains categorized as susceptible to the antibiotic considered only). European Committee on Antimicrobial Susceptibility Testing (EUCAST) thresholds for susceptibility (at standard or high doses) are as follows: ceftaroline: 1mg/L (threshold for pneumonia); ceftobiprole: 2mg/L; linezolid: 4mg/L; rifampicin: 0.5 mg/L; and trimethoprim: 4mg/L.

For chronically colonized patients, we searched for strains that had been isolated before the strains studied above, which met the following criteria: (i) strongly adherent strains/strains forming early biofilm as determined by the BRT® in filtered ASM (BFI≤4), and (ii) WGS-based analysis confirming the chronic colonization based on the identification of less than 123 SNPs between the sequences of the “early” and the “late” isolated strains (signing either a “same” strain or “very closely related” strains according to the criteria of Ankrum and Hall, 2017). A total of five earlier-isolated strains from four patients matching these criteria were selected and compared with six strains isolated during patient follow-up. The characteristics of patients and selected strains are presented in Figure 3A. MICs were determined, and an Antibiofilmogram® was performed on these strains (as described above), and bMICs are presented in Figure 3B. MIC and bMIC values for the five antibiotics were all below the EUCAST threshold for susceptibility for the five strains isolated earlier. For later-isolated strains that remained susceptible to antimicrobial agents in planktonic culture, we showed that ceftaroline and ceftobiprole (Patients 5 and 6) or linezolid (Patients 17 and 18) retained their ability to prevent biofilm formation (bMICs below the corresponding EUCAST thresholds for resistance) over time. We related these observations to the antimicrobial courses received by the patients (Figure 3). The more important antibiotic selective pressure was noted for linezolid and Patients 5 and 6. Three out of the four strains isolated in these patients were resistant to linezolid in planktonic cultures (resistance, investigated in a previous study; Boudet et al., 2021) was related to a G2576T substitution present in a variable number of 23S rRNA gene copies, while susceptible strain 6.3 displayed a high bMIC of 16mg/L. For strains isolated in Patients 17 and 18 (who had received fewer linezolid courses during their follow-up), increased bMICs were observed over time, but bMICs were still below the resistance threshold (Figure 3). Although a low number of rifampicin and trimethoprim courses (possibly associated) were noted in the four patients, both antibiotics were no more active on later-collected strains, showing MICs or bMICs above the corresponding resistance thresholds. None of the patients had received ceftaroline or ceftobiprole.

### Dynamics of Biofilm Formation Under Flow in the Presence and Absence of Antibiotics

**Figure 4:**
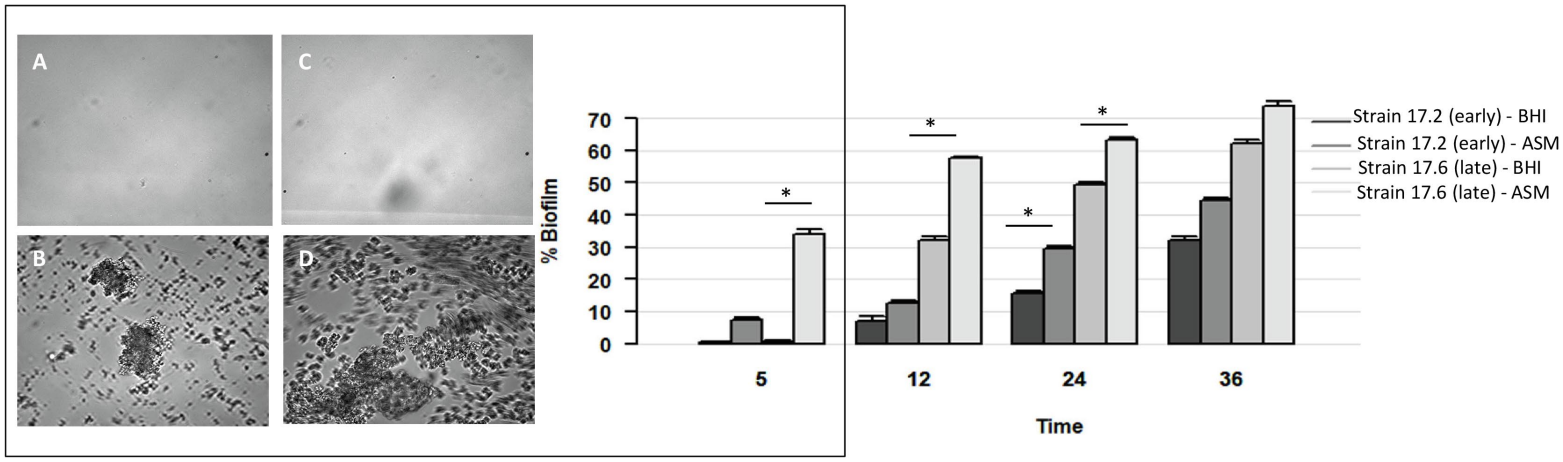
Dynamics of biofilm formation in BHI medium and ASM in the absence of antibiotics for two strains isolated 3years apart in Patient 17 (strain 17.2: “early” strain and 17.6: “late” strain). Left: Biofilm formation after 5h of incubation: **(A,B)** early strain 17.2 in BHI medium **(A)** and ASM **(B)**; **(C,D)** late strain 17.6 in BHI medium **(C)** and ASM **(D)**. Right. Percentage of biofilm formed over time (in hours). *p*-values are indicated for comparisons of biofilm formed by a strain depending on the culture medium at each time of the assay. ^*^Value of *p*<0.05 using *t* test.

To complete the characterization of biofilm formation of CF MRSA in the presence or absence of antibiotics, a study was performed in dynamic conditions using the BioFlux™ 200 microfluidic system. We selected strain 17.6, one of the two strains that were strongly adherent after 1.5h of incubation in the BRT® and the paired 17.2 strain isolated 3years before in Patient 17. For these paired strains, we first compared their dynamics of biofilm formation over time according to the medium used (BHI or ASM). The formation of biofilm increased regularly over time for each condition of the assay (“early” strain in BHI medium, “early” strain in ASM, “late” strain in BHI medium, “late” strain in ASM) with a systematically higher percentage of biofilm formed in ASM compared with BHI medium and for “late” strain compared with “early” strain (Figure 4). Although both strains were shown to have high adhesion ability using the BRT®, these results revealed that strains 17.2 and 17.6 each had distinct dynamics of biofilm formation, with a slower biofilm formation for the earlier-isolated strain 17.2 (Figure 4).

**Figure 5:**
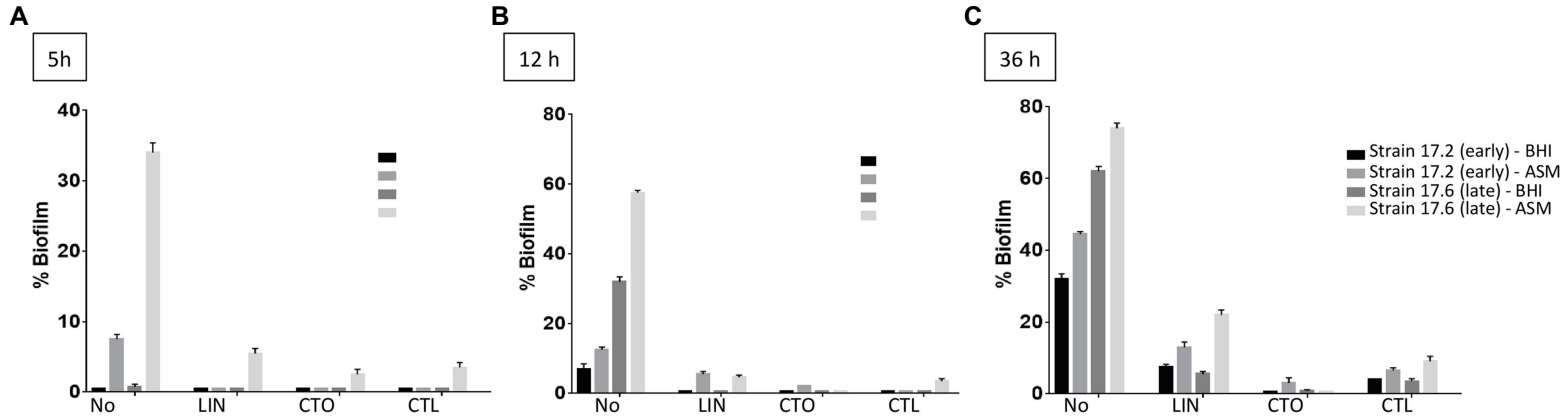
Dynamics of biofilm formation (% of biofilm formed) in BHI medium and ASM in the presence of antibiotics for two strains isolated 3years apart in Patient 17 (strain 17.2: “early” strain and 17.6: “late” strain). Results are presented comparatively to those observed in the absence of antibiotic previously presented in Figure 4. **(A)** After 5h of incubation; **(B)** after 12h of incubation; **(C)** after 36h of incubation. No, no antibiotic; LIN, linezolid; CTO, ceftobiprole; and CTL, ceftaroline. Antibiotics were added to a final concentration equivalent to the minimum biofilm inhibitory concentration (bMIC) in the corresponding medium as follows: strain 17.2, bMICs in BHI medium and in ASM: CTL: 0.125mg/L, CTO: 0.25mg/L, LIN: 0.5mg/L; strain 17.6, bMICs in BHI medium: CTL: 0.25mg/L, CTO: 0.5mg/L, LIN: 2mg/L; bMICs in ASM: CTL: 4mg/L, CTO: 4mg/L, and LIN: 2mg/L. Data are commented for statistical significance in the text.

Further testing with antibiotics was limited to three times of measure (5, 12, and 36h) and to the three antimicrobial agents with the lowest bMICs, that is, ceftaroline, ceftobiprole, and linezolid. With the BioFlux™ device, the percentage of biofilm formed in the presence of one of the three drugs tested was drastically lower than that observed for the control without antibiotics showing that all three antibiotics had the ability to limit biofilm formation and that this effect was long-lasting as it was observed up to 36h of exposure to the bMIC of the corresponding antimicrobial agent in both media (Figure 5). Differences in biofilm formation in ASM were compared for statistical significance by a t test. A significantly higher inhibition of biofilm formed by the earlier isolated strain 17.2 was observed from 12h of incubation and up to 36h of incubation for ceftaroline and ceftobiprole compared with linezolid (values of *p*<0.05 at 12h; values of *p*<0.01 at 36h). Differences observed between ceftaroline and ceftobiprole were also statistically significant (value of *p*<0.05 at 36h), ceftaroline being more active in biofilm formation inhibition than ceftobiprole after 12h, whereas ceftobiprole was more active in biofilm formation inhibition than ceftaroline after 36h. Inhibition of the biofilm formation by the “late” strain 17.6 in ASM was more pronounced with ceftobiprole than with linezolid (values of *p*<0.01 at 12 and 36h) or ceftaroline (values of *p*<0.01 at 36h). Ceftaroline was also more active than linezolid after 36h (value of *p*<0.01).

## Discussion

Cystic fibrosis is a chronic disease in which bacterial colonizations/infections of the airways are usual. Due to local specific conditions in the CF lung, diverse selective pressure is applied to colonizing microorganisms (interactions with the host immune response, high levels of antibiotic use, oxygen deprivation in mucus, altered antimicrobial peptide production, etc.) which, in turn, develop several traits to adapt to the surrounding environment and survive in the CF lung (Hauser et al., 2011). Among these traits, biofilm growth has been observed with several opportunistic pathogens in certain human diseases including CF, conferring protection against antimicrobial treatments and therefore contributing to bacterial persistence (Donlan and Costerton, 2002). While the role of biofilm and the dynamics of its formation have been extensively studied for *P. aeruginosa* (Høiby et al., 2010), there have been few studies on MRSA in CF patients. However, biofilm formation is an important virulence trait of *S. aureus* which has been frequently observed (67% of the strains) in a recent study on CF pediatric patients (Kodori et al., 2021). Biofilm formation was also significantly more often observed for strains isolated from CF patients compared with those recovered from non-CF patients (Aktas et al., 2013). In MRSA, biofilm represents an additional obstacle to eradication in addition to multidrug resistance making these strains the most problematic ones and a great matter of concern due to their involvement in negative clinical evolution (Ren et al., 2007; Dasenbrook et al., 2008; Vanderhelst et al., 2012). One recent study found that 85.6% of MRSA isolates were biofilm producers compared with 54.2% of MSSA, suggesting that MRSA isolates are better able to form biofilm during CF (Kadkhoda et al., 2020). Biofilm formation was also observed in 14 of 15 CF MRSA pulsotypes in the study by Molina et al. (2008), supporting the fact that biofilm formation is a common characteristic of CF MRSA. In the specific and distinct conditions of our study – BRT® used with a 4-h incubation for selection of strong producers of early biofilm rather than the microtiter test used in previous studies, we found a rate of strong biofilm-forming MRSA of 27% of the strains and these strains had been isolated in samples from 37% of the studied patients.

Previous studies have related the propensity of specific clones of *S. aureus* for forming biofilm (Vanhommerig et al., 2014; Tasse et al., 2018) as well as *Escherichia coli* (Flament-Simon et al., 2019). For CF MRSA, due to the lack of studies including genetically characterized strains, it remains to be explored whether some lineages are more prone to forming biofilm. More generally, there is a lack of knowledge of MRSA clones circulating in the CF population in Europe (Vu-Thien et al., 2010; Cocchi et al., 2011) although no comparisons can be drawn with non-European data, particularly American data, due to the highly distinct epidemiology between these continents (Glikman et al., 2008). Congruent with the studies of Vu-Thien et al. (2010) in France and Cocchi et al. (2011) in Italy, we observed that ST5 and ST8 were the most frequent STs identified in our study with 81% of the strains isolated in 80% of the patients belonging to these MLST genotypes. We observed that clonal lineages differed in terms of their biofilm-forming capacities as the majority of strong biofilm-producing strains belonged to ST5 and ST8, a trait that may contribute to the large representation of both genotypes in CF. However, due to the important representation of both STs in the overall population, ST5 and ST8 were major MLST genotypes observed in the three groups of BFI values in our study. They are also major MRSA lineages in the global population. Previous studies showed that some MRSA lineages like the predominant clone of community-acquired (CA)-MRSA in the United States, the USA300 clone in the clonal complex 8, have enhanced biofilm-forming capacity (Vanhommerig et al., 2014). Strains included in our study did not harbor the Panton-Valentine leukocidin-encoding gene characteristic of some CA-MRSA clones (unpublished) but were not characterized further in our study, thus preventing any conclusions about the implication of specific biofilm-forming lineages of ST5 or ST8 in CF.

Although antimicrobial treatments are considered as effective according to the results of *in vitro* assays, microorganisms may still persist in the CF airways for years, partly due to their ability to be protected within the polymer matrix produced during biofilm formation. In this context, the efficacy of antimicrobial therapy is reduced within the biofilm and approaches targeting bacterial biofilms in cystic fibrosis airways are required (Martin et al., 2021). However, as recently stated by Guzmán-Soto et al. (2021), “for the successful development of antibiofilm treatments and therapies, understanding biofilm development and being able to mimic such processes is vital.” Several natural or synthetic compounds have shown to be active on biofilm formed by *S. aureus* (Miquel et al., 2016). However, most studies on *S. aureus* focused on the effects of antibiotics on established biofilm in the context of biofilm-associated infections other than CF, mainly medical device-associated infections. The antibiofilm activity of antibiotics on *S. aureus* isolated from CF patients has been scarcely investigated. Considering the 5 antibiotics included in this work, a PubMed search conducted on July 19, 2021, with « ceftaroline » or « ceftobiprole » or « linezolid » or « rifampicin » or « trimethoprim » and « *Staphylococcus aureus* » and « cystic fibrosis » and « biofilm » found five non-redundant publications supporting the need for data acquisition on the subject of antibiotics’ antibiofilm activity against CF *S. aureus* isolates (Molina et al., 2008; Aktas et al., 2013; Iglesias et al., 2019; Gilpin et al., 2021; Harrington et al., 2021). This is reinforced by the observations that most of these antimicrobial agents have been found to display an antibiofilm activity on *S. aureus* isolated in other settings. For example, ceftaroline was effective against biofilm established by MRSA *in vitro* and *in vivo* models of catheter-associated biofilm formation suggesting that ceftaroline could be considered for the treatment of biofilm-associated MRSA infections (Meeker et al., 2016). In CF, ceftaroline was recently shown to represent an effective antimicrobial option for the management of acute pulmonary exacerbations involving MRSA in pediatric CF patients (Branstetter et al., 2020) and, on the other hand, has been shown to have an antibiofilm effect on biofilm-producing MRSA in other chronic infections (Mottola et al., 2016). Ceftobiprole has also previously demonstrated promising activity against biofilm from methicillin-resistant staphylococci (Abbanat et al., 2014). However, previous studies mainly included limited numbers of strains, either reference strains that may not reflect the behavior of CF strains or strains with no associated clinical data. In our study, we report the first data on the effect of antibiotics, including fifth-generation cephalosporins, on biofilm formation *via* a collection of clinically documented and genotypically characterized CF MRSA and seek to mimic CF sputum by using ASM in the different *in vitro* assays.

Due to the strong influence of environmental conditions on bacterial biofilm formation, the use of ASM formulated to mimic the sputum of CF patients appears promising for *in vitro* studies on CF isolates (Sriramulu et al., 2005; Dinesh, 2010). However, this technique remains little used in published studies and has mostly been used in studies on *P. aeruginosa* (Sriramulu et al., 2005; Kirchner et al., 2012; Rozenbaum et al., 2019; Iglesias and Van Bambeke, 2020). For *S. aureus*, it was previously used in a single study to determine the influence of the medium on the antibiofilm activity of antibiotics including two of those under consideration in this study (linezolid and rifampicin). ASM, containing amino acids, mucin, and free DNA, was shown to provide the most protective matrix (Rozenbaum et al., 2019) and all the antibiotics previously tested were drastically less potent and less effective in ASM than in comparators with respect to viability, metabolic activity, and biomass (Iglesias and Van Bambeke, 2020; Frisch et al., 2021). Here, through the comparative study of biofilm formation dynamics in ASM and BHI medium, we show that the use of ASM should be encouraged for further studies on biofilm formation by CF MRSA clinical isolates as an enhanced and accelerated biofilm formation was observed in ASM. However, we also found that ASM was not adaptable to all devices probably due to some of its characteristics like turbidity and viscoelasticity (Iglesias et al., 2019) and we had to consider filtered ASM for use in the BRT® despite the impact of the ASM filtration and the components which may be affected by this step remain unknown. Of note, ASM did not contain glucose despite glucose is present, at various levels according to the studies, in the sputum of CF patients (Van Sambeek et al., 2015; Nielsen et al., 2020) and glucose was shown to promote *S. aureus* biofilm formation (Lade et al., 2019). The supplementation of ASM with glucose may thus represent an interesting perspective for future work unless the best glucose concentration to be used can be defined considering the inter-individual (but also intra-individual) variability highlighted in previous studies of sputum samples from CF patients (Van Sambeek et al., 2015; Nielsen et al., 2020).

The inhibition of early biofilm formation observed in ASM in this study for linezolid and, more markedly, for ceftobiprole and ceftaroline represents an important finding because the first two stages of biofilm development, that is, adsorption and adhesion, are key determinants in the next stages leading to biofilm development during initial colonization by MRSA and also during the biofilm life cycle at the dispersal stage. These observations warrant complementary studies on established biofilm and on antibiotic combinations as previously studied in other settings such as endocarditis, diabetic foot infections, and medical device-associated infections for associations including ceftaroline (Barber et al., 2015; Mottola et al., 2016; Thieme et al., 2018), as they may contribute to the required optimization of antibiotic regimens against biofilm (Lebeaux et al., 2014).

Bacterial populations colonizing CF patients are known to be highly complex and dynamic, encompassing a variety of variants whose equilibrium varies according to the changing conditions of the surrounding environment. This population diversification is an increasingly well-known host adaptation strategy which has been widely studied in Gram-negative bacilli during chronic colonization of CF patients’ lungs (Winstanley et al., 2016; Menetrey et al., 2021). For *S. aureus*, diversified populations have been more rarely documented in CF (Goerke et al., 2007; Vu-Thien et al., 2010; Hirschhausen et al., 2013; Ankrum and Hall, 2017; Boudet et al., 2021; Wieneke et al., 2021). Regarding biofilm formation, we observed that patients may be colonized by clonally related strains corresponding to adaptive variants displaying distinct abilities to form biofilm, as previously described (Savage et al., 2013; Wieneke et al., 2021). Such diversification of MRSA populations and intra-sample heterogeneity of biofilm formation ability may contribute to compromising the antibiotic management of CF airway infections. Other adaptive phenotypic modifications observed for CF *S. aureus* have included the increase in antimicrobial resistance and the biofilm development observed in certain studies (Hirschhausen et al., 2013). In our study, strains adapted to the CF lung environment after years of colonization may still display low bMICs for ceftaroline, an observation that requires further investigation on a larger panel of MRSA strains isolated in chronically colonized patients. Adaptive modifications like mucoidy and nuclease activity were recently shown to be more frequently observed in case of *P. aeruginosa* co-colonization highlighting the importance of cross-talk and interactions between bacterial pathogens in shaping the adaptation of *S. aureus* to the CF lung environment (Wieneke et al., 2021). In our study, no obvious association was observed between chronic colonization by *P. aeruginosa* and the ability of MRSA to form early biofilm. Finally, future studies are still needed to investigate the behavior of CF MRSA within multispecies biofilm as well as the effect of exposure of these multispecies biofilms to different antibiotics (Vandeplassche et al., 2020).

## Conclusion

We hereby report the first data on early biofilm formation by MRSA clinical isolates from CF patients using original and standardized approaches which had never been previously applied to such isolates and whose use should be encouraged. Indeed, the BRT® is well-designed to investigate the early and capital stages of biofilm formation contrarily to the most frequently used microtiter plate dye-staining (crystal violet) assays and presents the other advantages of being a rapid, high-throughput, easy-to-handle, and highly reproducible method. The BioFlux is meanwhile a fully integrated and easy-to-use system allowing the study of the dynamic formation of the biofilm and the small volumes required make this system highly applicable for screening of biofilm inhibitory agents (for a critical review of methods usable to study biofilm formation, see Azeredo et al., 2017).

As previously observed for other CF pathogens like *P. aeruginosa* or in other settings like medical device-associated infections, antibiotic susceptibility was reduced in biofilm, thereby complicating anti-MRSA treatments and representing a risk of treatment failure in CF patients. The Antibiofilmogram® is a promising tool for guiding the choice of the most effective drugs against biofilm formation by MRSA in CF airways. In our study, the two broad-spectrum anti-MRSA cephalosporins ceftaroline and ceftobiprole displayed a notable ability to limit biofilm formation of CF MRSA. Hitherto little used for CF patients, the characteristics of both antimicrobial agents, together with recent studies showing that ceftaroline represents an additional treatment option for treating MRSA-associated acute pulmonary exacerbations (Branstetter et al., 2020), should promote their larger use in the management of CF patients infected by MRSA.

## Data Availability Statement

The datasets presented in this study can be found in online repositories. The names of the repository/repositories and accession number(s) can be found in the article/Supplementary Material.

## Ethics Statement

The studies involving human participants were reviewed and approved by the Institutional Review Board at Nîmes University hospital (Interface Recherche Bioéthique IRB n 21.03.01, March 04, 2021). Written informed consent from the participants’ legal guardian/next of kin was not required to participate in this study in accordance with the national legislation and the institutional requirements.

## Author’s Note

This work was partly presented at the 44th European Cystic Fibrosis Conference (digital), 9–12 June 2021.

## Author Contributions

HM: conceptualization. AB, PS, and CP: methodology. AB, PS, CP, CD-R, and HM: formal analysis. AB, PS, and RC: investigation. AB, PS, CP, and RC: data curation. AB, PS, and HM: writing – original draft preparation. CP, CD-R, RC, and J-PL: writing – review and editing. AB, CD-R, and HM: supervision. CD-R, J-PL, and HM: funding acquisition. All authors contributed to the article and approved the submitted version.

## Funding

This research was funded by the University Hospital of Nîmes (NîmAO 2018.02 grant).

## Conflict of Interest

The authors declare that this research was conducted in the absence of any commercial or financial relationships that could be construed as potential conflicts of interest.

## Publisher’s Note

All claims expressed in this article are solely those of the authors and do not necessarily represent those of their affiliated organizations, or those of the publisher, the editors and the reviewers. Any product that may be evaluated in this article, or claim that may be made by its manufacturer, is not guaranteed or endorsed by the publisher.

## Footnotes

^1^ https://pubmlst.org/saureus/
