# Supplementary material for: Biofilm Formation in Methicillin-Resistant Staphylococcus aureus Isolated in Cystic Fibrosis Patients Is Strain-Dependent and Differentially Influenced by Antibiotics
Source: Front Microbiol. 2021 Oct 15;12:750489. doi: 10.3389/fmicb.2021.750489 (PMC8554194; doi:10.3389/fmicb.2021.750489)
Supplement: Supplementary file 1 [file Table_1.DOCX]

Supplementary Material

# 1 Supplementary Table. Patients and characteristics of the CF MRSA collection.

| **Patient designation** (chronic colonization*) | **Year of birth, sex** | **Year of first MRSA colonization** | **MRSA strain designation**** | **Year of isolation** | **ST** | **Average BFI** (BRT®, 4h) | **BFI group***** | **Co-colonizing pathogens** (CC) |
| --- | --- | --- | --- | --- | --- | --- | --- | --- |
| 1 (CC) | 1988, M | NA | 1.1 | 2014 | 8 | 4.00 |  | *Pa* |
| 2 (CC) | 2000, F | 2002 | 2.1 | 2009 | 572 | 10.8 |  | *Pa* |
|  |  |  | 2.2 |  | 572 | 20.30 |  |  |
|  |  |  | 2.3 |  | 572 | 20.11 |  |  |
| 3 (CC) | 1967, M | 2012 | 3.4 | 2016 | 5 | 11.68 |  | - |
|  |  |  | 3.5 |  | 5 | 20.31 |  |  |
| 4 | 1990, M | 2010 | 4.1 | 2016 | 5 | 8.78 |  | *Pa* |
| 5 (CC) | 1996, F | 2003 | 5.2 | 2016 | 5 | 0.12 |  | *Pa* |
|  |  |  | 5.3 |  | 5 | 0.75 |  |  |
| 6 (CC) | 1991, F | 2003 | 6.3 | 2016 | 5 | 0.58 |  | *Pa* |
|  |  |  | 6.4 |  | 5 | 0.25 |  |  |
| 7 | 2005, F | 2007 | 7.2 | 2016 | 5 | 0.71 |  | - |
| 8 | 1988, M | NA | 8.1 | 2015 | 34 | 20.3 |  | - |
| 9 | 1982, M | 2006 | 9.1 | 2016 | 8 | 20.27 |  | *Pa* |
| 10 (CC) | 1984, M | NA | 10.4 | 2016 | 8 | 20.26 |  | *Pa* |
|  |  |  | 10.5 |  | 8 | 20.23 |  |  |
|  |  |  | 10.6 |  | 8 | 20.26 |  |  |
| 11 | 2002, F | 2013 | 11.3 | 2016 | 5 | 20.29 |  | *Pa* |
|  |  |  | 11.4 |  | 5 | 20.27 |  |  |
|  |  |  | 11.5 |  | 5 | 20.30 |  |  |
| 12 | 2002, F | 2007 | 12.1 | 2007 | 5 | 11.61 |  | *Ax* |
| 13 | 1998, F | 2014 | 13.1 | 2014 | 5 | 20.18 |  | - |
| 14 | 1983, M | 2008 | 14.3 | 2014 | 5 | 20.27 |  | *Pa*, *Ma* |
|  |  |  | 14.4 |  | 4782 | 20.28 |  |  |
| 15 | 1989, M | NA | 15.1 | 2016 | 4886 | 20.11 |  | *Pa*, *Bc* |
| 16 (CC) | 1990, F | NA | 16.2 | 2014 | 5 | 20.27 |  | - |
| 17 (CC) | 1996, F | 2001 | 17.4 | 2016 | 5 | 10.8 |  | *Pa* |
|  |  |  | 17.5 |  | 5 | 8.37 |  |  |
|  |  |  | 17.6 |  | 5 | 0 |  |  |
| 18 (CC) | 1981, F | 2009 | 18.2 | 2016 | 8 | 3.03 |  | *Pa* |
|  |  |  | 18.3 |  | 8 | 11.77 |  |  |

# Supplementary Table (to be continued).

| 19 | 1983, M | NA | 19.1 | 2016 | 30 | 20.28 |  | *Pa*, *Ma* |
| --- | --- | --- | --- | --- | --- | --- | --- | --- |
|  |  |  | 19.2 |  | 30 | 20.25 |  |  |
| 20 | 1991, F | NA | 20.1 | 2015 | 5828 | 0.66 |  | *Pa* |
| 21 (CC) | 1999, F | 2005 | 21.2 | 2016 | 5 | 8.47 |  | - |
|  |  |  | 21.3 |  | 5 | 9.165 |  |  |
| 22 (CC) | 1964, F | 2005 | 22.5 | 2016 | 8 | 12.20 |  | *Ax* |
|  |  |  | 22.6 |  | 8 | 19.87 |  |  |
|  |  |  | 22.7 |  | 8 | 13.52 |  |  |
| 23 (CC) | 2014, M | 2014 | 23.3 | 2016 | 8 | 20.24 |  | - |
| 24 | 1996, M | NA | 24.1 | 2016 | 5 | 9.20 |  | *Ax* |
|  |  |  | 24.2 |  | 5 | 10.85 |  |  |
| 25 (CC) | 1988, F | NA | 25.5 | 2016 | 5 | 4 |  | - |
|  |  |  | 25.6 |  | 5 | 3.22 |  |  |
|  |  |  | 25.7 |  | 5 | 20.20 |  |  |
|  |  |  | 25.8 |  | 5 | 20.20 |  |  |
| 26 | 1982, M | 2007 | 26.1 | 2016 | 8 | 2.08 |  | *Pa* |
|  |  |  | 26.2 |  | 8 | 10.20 |  |  |
| 27 (CC) | 1973, F | 2004 | 27.2 | 2016 | 8 | 20.28 |  | *Pa* |
|  |  |  | 27.4 |  | 8 | 20.18 |  |  |
|  |  |  | 27.5 |  | 8 | 8.80 |  |  |
| 28 (CC) | 2005, F | 2015 | 28.3 | 2016 | 5 | 0 |  | *Pa* |
|  |  |  | 28.4 |  | 5 | 0.25 |  |  |
|  |  |  | 28.5 |  | 5 | 12.84 |  |  |
|  |  |  | 28.6 |  | 5 | 10.55 |  |  |
| 29 | 1986, M | 2005 | 29.1 | 2016 | 5 | 20.16 |  | *Pa* |
| 30 | 1983, M | NA | 30.1 | 2016 | 5 | 0 |  | *Pa* |
| 31 (CC) | 1998, F | 2005 | 31.2 | 2016 | 8 | 2.68 |  | *Pa* |
| 32 | 2005, M | NA | 32.1 | 2016 | 8 | 1.89 |  | - |
| 33 | 1998, F | NA | 33.1 | 2015 | 8 | 20.19 |  | *Sm* |
|  |  |  | 33.2 |  | 5829 | 20.20 |  |  |
| 34 | 2004, F | NA | 34.1 | 2015 | 30 | 20.27 |  | - |
| 35 | 1986, M | 2009 | 35.2 | 2015 | 239 | 11.61 |  | *Pa*, *Pp* |

* chronic MRSA colonization at the time of strain inclusion in the study.

** multiple strains from the same patient correspond to distinct colony morphotypes co-cultured from one sputum sample.

*** Biofilm Formation Index groups: light grey, BFI of 19.87 to 20.31 (non-adherent strains); intermediate grey, intermediate BFI values comprised between 8.37 and 13.52; dark grey, BFI ≤4 (early biofilm formers).

CC = chronic colonization.

M = male; F = female.

NA = not available.

BFI = Biofilm Formation Index.

BRT = BioFilm Ring Test® (results obtained after a 4-h incubation in 0.22 mm filtered Artificial Sputum Medium.

*Pa*, *Pseudomonas aeruginosa*; *Ax*, *Achromobacter xylosoxidans*; *Ma*, *Mycobacterium abscessus*; *Bc*, *Burkholderia cenocepacia*; *Sm*, *Stenotrophomonas maltophilia*; *Pp*, *Pandoraea pulmonicola*.

# 2 Supplementary Figures

## 2.1 Supplementary Figure 1

##

**Supplementary Figure 1.** Comparison of bacterial growth of three CF MRSA strains in BHI medium, ASM and 0.22µm filtered-ASM over a 24-hour growth period.

**A.** Strain 5.3. **B.** Strain 6.4. **C.** Strain 17.6.

Growth curves present the number of Colony Forming Units (CFU) in log10.ml-1 according to the time of incubation at 37 °C (in hours).

Blue, Brain Heart Infusion (BHI) medium; orange, Artificial Sputum Medium (ASM); grey, 0.22µm filtered- ASM.

**2.2 Supplementary Figure 2**

**Supplementary Figure 2.** Comparison of Biofilm Formation Index determined by the BioFilm Ring Test® according to the medium for 2 CF MRSA strains after a 4h-incubation at 37 °C.

BHI, Brain Heart Infusion; ASM, Artificial Sputum Medium.

****P*-value <0.001 by Mann–Whitney U test.

**2.3 Supplementary Figure 3**


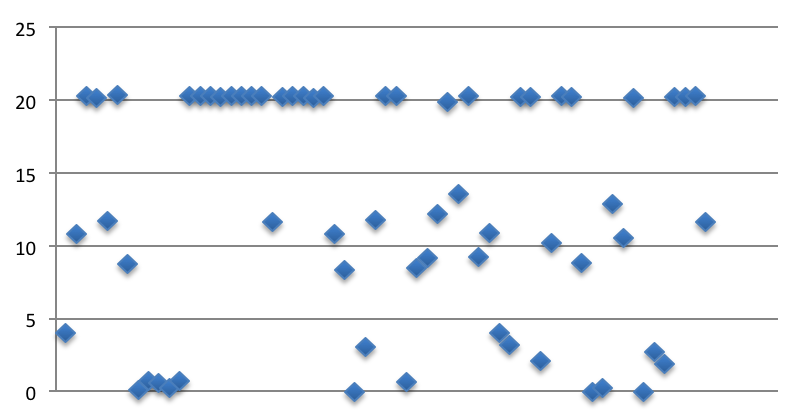


**BFI value**

Non-adherent

(BFI: 19.87 to 20.31)

Early biofilm formers

(BFI: ≤4)

Intermediate group

(BFI: 8.37 to 13.52)

**Supplementary Figure 3.** Distribution of the Biofilm Formation Index (BFI) values for the 63 CF MRSA strains of the study supporting the classification of the isolates in 3 groups according to their ability to form early biofilm.
